# Golf‐Related Injuries in Young Elite Golfers: A Cross‐Sectional Survey Study

**DOI:** 10.1002/hsr2.72333

**Published:** 2026-04-16

**Authors:** Stephen Lee, Michele Lastella, Andrew Vitiello, Henry Pollard

**Affiliations:** ^1^ School of Health, Medical, and Applied Sciences Central Queensland University Perth Western Australia Australia; ^2^ Appleton Institute for Behavioural Science Central Queensland University Adelaide South Australia Australia; ^3^ Private Practice Sydney New South Wales Australia; ^4^ Central Queensland University Rockhampton Queensland Australia; ^5^ Faculty of Health Sciences Durban University of Technology Durban South Africa

**Keywords:** adolescent, child, golf, injury, young adult

## Abstract

**Objectives:**

This study aims to investigate the prevalence, patterns, and management strategies of golf‐related injuries among elite adolescent golfers, while also examining the relationship between training volume and injury occurrence.

**Design:**

Cross‐sectional survey study.

**Method:**

The study involved elite adolescent golfers (*n* = 56, response rate 73%) aged 10–19 years with a golf handicap index of ≤ 19, located in Western Australia. An electronic survey (Qualtrics) was utilised to collect retrospective data on injuries and training volume over the preceding 12 months. Descriptive statistics were employed to summarise the frequencies and proportions of participant responses, while logistic regression analyses were conducted to estimate odds ratios and confidence intervals, assessing associations between participant characteristics, training practices, and injury risk.

**Results:**

The 12‐month injury incidence rates were 0.229, 0.624, and 0.100 per 1000 h dedicated to golf practice, competition, and their aggregation, respectively. The primary injury locations identified were the mid back (47.06%), ankle (29.41%), lower back (29.41%), and knee (23.52%). A total of 17 participants (30.36%) reported at least one injury, with 3 (17.65%) being recurrences. Of the documented injuries, 8 (47.00%) were classified as golf‐related, while 9 (52.94%) resulted from other activities. No significant correlation was found between training volume and injury risk (OR: 0.96; 95% CI: 0.87–1.06).

**Conclusions:**

Elite adolescent golfers exhibited 30.36% injury prevalence with an incidence rate of 0.100 per 1000 h. Mid‐back (47.06%), ankle (29.41%), and lower back (29.41%) were most affected. Training volume showed no significant injury association (OR: 0.96; 95% CI: 0.87–1.06), possibly due to limited sample size.

## 1 Introduction

Golf has experienced significant growth in Australia, particularly among adolescents [1]. Data from Golf Australia revealed a 16% rise in junior membership, with over 24,000 new club members nationally between 2020 and 2021 [1]. The sport is witnessing younger participants achieving peak performance rapidly and securing substantial financial gains [2]. Recently, youthful talent on the PGA Tour has set new records and competed at elevated standards, achieving professional victories in their early twenties [2].

The increasing participation of adolescents in elite sports has raised interest in the epidemiology of sports‐related injuries among youth, including golf [3]. Although golf is often seen as low‐impact, elite players face unique injury risks due to repetitive motions, particularly in the spine, wrists, elbows, and shoulders [4]. Injury rates are driven by the biomechanical demands of the sport, such as high torque and range of motion in the golf swing [5]. Research suggests injury incidence may be underreported, with lumbar spine and upper extremity injuries being the most prevalent among elite youth golfers, paralleling trends in older players [4, 6].

Studies on golf‐related injuries primarily focus on adult populations [7]. A recent systematic review and meta‐analysis by Kuitunen and Ponkilainen (2024) synthesised injury incidence data across golf populations and identified a critical gap in the literature, highlighting that only seven studies met inclusion criteria for meta‐analysis, and research specifically examining injury patterns in adolescent golfers remains severely limited [8]. A scoping review indicated that while the overall injury rate in golf is low, specific data on adolescent golfers is scarce, often aggregating data across a wide age range [7]. Further, current data does not adequately characterise the injury profile of elite adolescent golfers, mainly focusing on injuries related to golf carts, balls, or clubs rather than those incurred during play [7]. Therefore, while research into prevention of golf injuries for adults is limited, there is virtually no data on adolescent golfers [3]. This study seeks to bridge that gap by establishing epidemiological data and systematically investigating the locations, frequencies, management strategies, and risk factors for injuries in elite adolescent golfers. Understanding the effects of training volume and injury patterns in this group could lead to targeted interventions and inform guidelines aimed at reducing injury incidence, improving player longevity, and enhancing safety in youth sports [9].

The researchers hypothesise that injury patterns in elite adolescent golfers will resemble those of amateur adult golfers, with common injuries including low back pain, elbow pain, and shoulder pain, more likely occurring in older male athletes. Further, older age, less golf experience, participation in other sports, and high training workloads may correlate with a higher injury rate. Lastly, weekly golf tuition, a regular warm up routine, and a minimum of two conditioning sessions per week are likely associated with reduced injury rates compared to elite adolescent golfers not completing these activities.

## 2 Methods

### 2.1 Definitions

‘Adolescent’ was delineated as individuals whose age ranged between 10 and 19 years, as defined by the World Health Organization. A formal definition of ‘injury’ and a differentiation between a ‘golf‐related injury’ and a ‘non‐golf‐ related injury’ were not provided to participants to maintain clarity, particularly given the adolescent age range of 10 to 19 years. While this may have increased variability in participants' interpretations, standardised definitions were omitted to reduce participant burden.

### 2.2 Participants

A total of 77 male and female participants between the ages of 10 to 19 years were invited to participate in this cross‐sectional study and complete an online survey in May 2024. All 77 participants were selected by Golf Western Australia (Golf WA) and classified as ‘elite’ due to their selection for the Talent Development Program. This program is designed to facilitate the advancement of athletes who are eligible for both current and prospective junior state squads, utilising golf stroke averages as a criterion for selection. Parental consent was secured to permit the inclusion of the participant in the study.

### 2.3 Survey Instrument

The survey consisted of 58 multiple choice questions pertaining to age, gender, handicap, golf experience, handedness, warm‐up habits, strength and conditioning activities, golf‐related practice and play, participation in other sports, golf instruction, equipment fitting, and injury status over the preceding 12 months. In instances where injuries were reported, participants were asked to locate the site of their injury based on a body diagram provided. Supplementary questions were then posed to ascertain whether the injury was golf‐related, its implications for golf practice and/or performance, and whether treatment was pursued. Participants were subsequently requested to enumerate their three most significant injuries from the prior year, and regarding their most severe injury, they were asked to elaborate on aspects including injury onset, mechanism, and prior history. Text entry was required for some questions to provide further detail when ‘Other’ was selected.

### 2.4 Procedure

The online survey was hosted on Qualtrics (Qualtrics. (2024). Qualtrics XM [Software]. Provo, Utah, USA. https://www.qualtrics.com) and disseminated via text message and email. Initial distribution was aligned with the end‐of‐year Talent Development Program Camp with a response window of 8 weeks. Participants received follow‐up reminder messages and emails at one, three, and 7 weeks subsequent to the initial distribution in accordance with the Dillman Total Design Method [10].

### 2.5 Ethical Considerations

Prior to survey distribution, an informational document was emailed to prospective participants and their guardians, outlining potential benefits, risks, confidentiality protocols, the consent process, and support services. Participants were assured of anonymity and that their choice to engage would not influence their affiliation with Golf WA or the research team. Results would be identifiable only by the researcher and not accessible to others. Upon completion, aggregated results would be shared via email, with individual results available upon request. All data would be securely archived within Central Queensland University's research storage. Parental consent was obtained to include participants in the study. Ethics approval was granted by the Human Research Ethics Committee of the Research Division at Central Queensland University under application reference 0000022280.

### 2.6 Analysis Plan

Frequencies and proportions of participant responses to relevant survey items were calculated using RStudio (packages: ggplot2; dplyr; tidyr; flextable; and officer) (Rstudio Team, 2024) to describe the incidence and patterns of golf‐related and non‐golf‐related injuries within the cohort, and to evaluate treatment and management practices concerning reported injuries. To evaluate the correlation between specific player characteristics and injury incidence, chi‐square tests and logistic regression analyses were performed using base packages in R Studio. Chi‐square statistics and corresponding *p*‐values were calculated to investigate relationships between injury incidence and binary independent variables. *p*‐values less than 0.05 were deemed statistically significant. Logistic regression analyses were employed to derive odds ratios (OR) and 95% confidence intervals (95%CI) to determine associations of continuous or multi‐level ordinal variables with injury occurrence. Odds Ratios were considered statistically significant if the 95% confidence interval did not include 1.00. When anticipated cell counts were below 5 (notably for ‘golf‐related’ injuries), Fisher's exact tests and exact logistic regression methods were employed.

### 2.7 Quality Assurance

The STROBE checklist for cross‐sectional studies was used to guide reporting for this research study. Injury prevalence will be reported in number of injuries per 1000 player hours so that it may be directly compared with other epidemiological studies of sporting activity as recommended in the International Olympic Committee consensus statement [11].

## 3 Results

### 3.1 Demographic Information

A total of 56 golfers (31 males, 25 females) participated in the survey, yielding a response rate of 73%. Participants were predominantly aged 13 to 15 years (64.29%, *n* = 36), with 25.00% (*n* = 14) aged 16 or older. Nearly half (48.21%, *n* = 27) had mid to high golf handicaps (7–19), 25.00% (*n* = 14) had low handicaps (1–6), 7.14% (*n* = 4) were scratch or above, and 19.64% (*n* = 11) were unclassified. Most identified as right‐handed (87.50%, *n* = 49), with 8.93% (*n* = 5) left‐handed and 3.57% (*n* = 2) ambidextrous. In terms of swing handedness, the majority were right‐handed (94.64%, *n* = 53).

### 3.2 Golf‐Related Training and Play

#### 3.2.1 Warm up

A significant number of participants (64.29%, *n* = 36) consistently engaged in warm‐up exercises before playing golf, while 32.14% (*n* = 18) occasionally warmed up, and 3.57% (*n* = 2) did not. Predominant warm‐up methods included hitting balls (85.71%, *n* = 48), practice air swings (69.64%, *n* = 39), stretches (66.07%, *n* = 37), and range of motion exercises (48.21%, *n* = 27).

#### 3.2.2 Strength and Conditioning

A total of 80.36% of participants (*n* = 45) indicated engagement in golf‐related strength and conditioning training, including strength and/or stretching sessions. Fourteen participants (31.11%) performed strength exercises twice weekly, 24.44% (*n* = 11) once weekly, and 22.22% (*n* = 10) three times weekly. Strength training sessions predominantly lasted 30 to 60 min (54.05%, *n* = 20), with 16.22% (*n* = 6) dedicating 15 to 30 min. Golf‐related stretching was more frequent but shorter in duration, with 24.44% (*n* = 11) stretching twice weekly, 22.22% (*n* = 10) three times weekly, 20.00% (*n* = 9) four or more times, 11.11% (*n* = 5) once weekly, and 22.22% (*n* = 10) not stretching at all. Stretching sessions mostly ranged from 5 to 15 min (45.71%, *n* = 16) or 15 to 30 min (37.14%, *n* = 13).

#### 3.2.3 Golf Practice and Playing Golf

Participants reported an average of 6.46 (SD = 4.17) practice rounds of nine holes and 8.53 (SD = 3.69) competition rounds monthly (where 18 holes equals 2 rounds). The standard time to complete 9 holes during practice was 1.5 h or less (44.64%, *n* = 25), while 41.07% (*n* = 23) indicated 2 h. In competition, half of the participants (50.00%, *n* = 28) completed nine holes in 2 h, and 37.50% (*n* = 21) needed 2.5 h.

### 3.3 Participation in Other Activities

A total of 36 participants (64.29%) indicated engagement in at least one additional sport, with basketball (36.11%) and swimming (27.78%) being the most common. Involvement in these sports ranged from 0 to 5 h per week (M = 1.88; SD = 1.23) and spanned 0 to 12 months annually (M = 6.23; SD = 4.15).

### 3.4 Golf Tuition and Club Fitting

Fifty participants (89.29%) indicated they had received instruction from a golf professional, primarily on a weekly (*n* = 20, 40.82%) or biweekly (*n* = 20, 40.82%) basis for either 30 min (*n* = 24, 48.98%) or 1 h (*n* = 20, 38.78%). Additionally, 43 participants (78.18%) reported undergoing club fitting, with 32.56% (*n* = 14) fitted once and 37.21% (*n* = 16) fitted twice.

### 3.5 Injuries

A total of 17 individuals (30.36%) reported at least one injury in the previous year, with 16.36% (*n* = 9) experiencing a singular injury and 14.55% (*n* = 8) indicating 2–4 injuries. Among the injured, 5 (8.92%) specified that the injury occurred during golf practice or play, accounting for a total of 8 golf‐related injuries. The predominant injuries included mid back (*n* = 8, 47.06%), ankle (*n* = 5, 29.41%), lower back (*n* = 5, 29.41%), and knee injuries (*n* = 4, 23.52%).

Eight injuries were golf‐related, resulting in annual incidence rates of 0.229, 0.624, and 0.100 per 1000 h for practice, competition, and combined activities, respectively. This corresponds to a mean of 0.004, 0.011, and 0.002 injuries per individual for every 1000 h of practice, competition, and aggregate activities, respectively (refer to Table 1). Golf‐related practice includes practice rounds, simulator sessions, driving range practice, putting, chipping, bunker shots, lessons, and strength and stretching exercises.

**Table 1:** Aggregated and per person annual 1000 h injury rates.

	Practice	Competition	Combined
Aggregated			
Golf injury	0.229	0.624	0.100
All injuries	0.946	2.574	0.412
Per person			
Golf injury	0.004	0.011	0.002
All injuries	0.017	0.046	0.007

**Figure 1:**
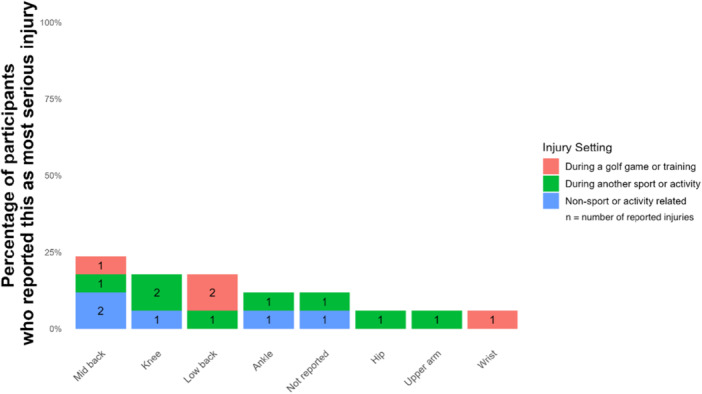
Most serious injuries according to when they were sustained.

**Figure 2:**
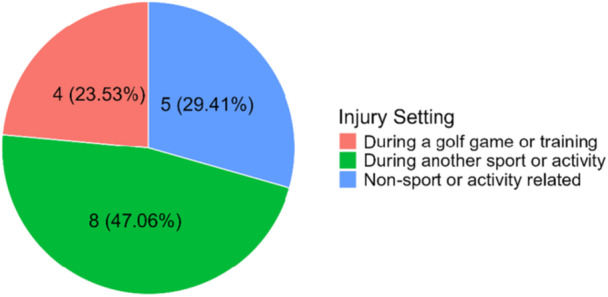
Pie chart of when most serious injury occurred.

Participants then elaborated on their most significant injury in the past year. Among the 17 individuals, 4 (26.67%) were mid back injuries and 3 (17.64%) were knee injuries. Figure 1 shows the timing of these injuries, while Figure 2 illustrates that most (*n* = 8, 47.06%) occurred during other sports. Of these, 3 were attributed to basketball, 2 to running, 1 to football, and 1 to a pregnancy‐related incident. Three injuries (17.65%) were recurrences; 2 occurred 1 to 2 years ago, and 1 over 5 years ago. Seven individuals indicated their injury was exacerbated during golf (*n* = 7, 41.18%), while 9 reported aggravation from other activities (*n* = 9, 52.94%), including basketball (*n* = 3) and running (*n* = 2).

### 3.6 Factors Associated With Injury Risk

Chi‐square analysis and Fisher's exact tests evaluated potential correlations between injury risk and factors such as gender, warming up, engagement in other sports, receiving professional instruction, and club fitting. The same methods assessed whether age (OR: 1.23; 95% CI: 0.94–1.66), frequency of practice (OR: 1.02; 95% CI: 0.81–1.29) or competition (OR: 1.14; 95% CI: 0.91–1.50), or total training volume (OR: 0.96; 95% CI: 0.87–1.06) influenced injury incidence. No statistically significant relationships were identified between these factors and the likelihood of reporting an injury in the preceding year. (See Table 2).

**Table 2:** Chi square and Fisher's exact test results.

	Any injury in past year			
	Yes	No	*χ* ^2^	*p*
Regular warm up			0.25	.62
Yes	35.00%	71.43%		
No	28.57%	65.00%		
Plays other sport				
Yes	28.57%	71.43%	0.25	.62
No	35.00%	65.00%		
Gender				
Male	30.00%	70.00%	0.30	.87
Female	32.00%	68.00%		
			**OR**	***p***
Professional tuition			1.13	1.00
Yes	33.33%	66.66%		
No	30.61%	79.39%		
Fitted clubs			0.69	.73
Yes	25.00%	75.00%		
No	32.55%	67.45%		

	Golf injury in past year			
	Yes	No	OR	*p*
Regular warm up			2.34	.65
Yes	11.11%	88.89%		
No	5.00%	95.00%		
Plays other sport			2.94	.34
Yes	5.56%	94.44%		
No	15.00%	85.00%		
Gender			0.81	1.00
Male	9.68%	90.32%		
Female	8.00%	92.00%		
Professional tuition			2.26	.45
Yes	16.67%	83.33%		
No	8.00%	92.00%		
Fitted clubs			2.61	.28
Yes	16.67%	83.33%		
No	6.98%	93.02%		

*Note:* OR = Odds ratio (i.e., from Fisher's exact test).

### 3.7 The Effect of Injuries on Golf Performance

Of the twelve individuals with non‐golf related injuries, nine (75.00%) reported an impact on their golf performance. Adjustments included missing competitions (*n* = 8, 14.29%) and reduced practice time (*n* = 7, 12.50%) as shown in Table 3.

**Table 3:** Modifications made to golf practice when injured.

	Count	(%)
Skipped competition	8	14.29
Decreased practice time	7	12.50
Could not finish competition	1	1.79
Other	1	1.79

### 3.8 Management of Injuries

**Figure 3:**
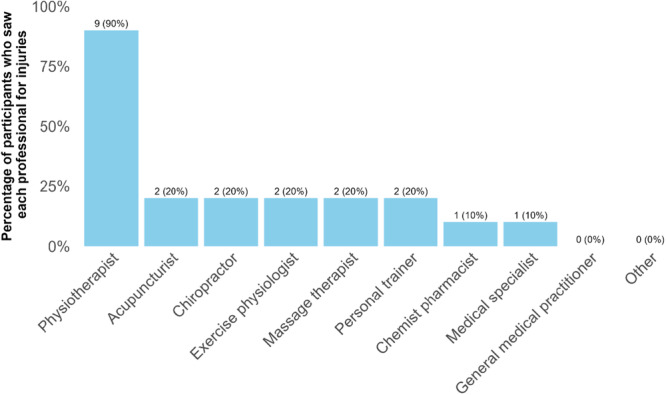
Treatments sought for non‐golf related injuries.

Among the five participants with a golf‐related injury in the past year, only two sought therapeutic intervention; one received treatment from multiple professionals, while the other consulted a physiotherapist. Of the twelve participants with non‐golf‐related injuries, ten engaged with a professional for treatment. As shown in Figure 3, nine participants (90.00%) sought physiotherapy, while two consulted other professionals. Only one reported treatment from a medical specialist, and no participants sought help from a general practitioner or alternative sources. Treatment from a physiotherapist was the most common choice among those detailing their most severe injury.

## 4 Discussion

In recent years, the prevalence and nature of injuries among adolescent athletes have garnered increasing attention, yet the specific context of elite adolescent golfers remains underexplored. This survey study aimed to illuminate the injury patterns within this demographic, which has historically received limited focus in the sports medicine literature.

### 4.1 Injury Rates and Injury Profile

Among elite adolescent golfers, our findings revealed an annual incidence rate of 0.229, 0.624, and 0.100 per 1000 h dedicated to golf practice, competition, and their aggregation, respectively. This translates to a mean of 0.004, 0.011, and 0.002 injuries per individual for every 1000 h of practice, competition, and combined activities. To contextualize these findings within the broader golf injury literature, it is instructive to compare our results with the recent systematic review and meta‐analysis by Kuitunen and Ponkilainen (2024), which reported a pooled injury incidence of 2.5 per 1000 athlete exposures (18 holes) across all golf populations [8]. Converting our combined incidence rate of 0.100 per 1000 h to the standardized metric of per 1000 athlete exposures (assuming 4 h per 18‐hole round) yields approximately 0.4 injuries per 1000 athlete exposures. This rate is substantially lower than the meta‐analytic pooled estimate and more closely approximates the incidence observed in amateur golfers (1.3 per 1000 exposures) rather than professionals (8.5 per 1000 exposures) [8]. This alignment with amateur rates, despite our participants being classified as elite adolescents, may reflect the protective effect of younger age and lower cumulative exposure compared to adult professional populations. However, direct comparisons are complicated by the systematic review's acknowledgment that only seven studies were available for meta‐analysis, with limited representation of adolescent cohorts specifically [8]. This reinforces the novelty of the current investigation and highlights the critical need for age‐stratified injury surveillance to establish normative injury rates across developmental stages in golf.

The substantially lower injury incidence observed in the current study becomes even more apparent when compared to recent data from adult amateur golfers. Williams et al. (2024) reported an overall injury incidence of 41.26 per 1000 h among predominantly amateur UK‐based golfers (*n* = 303), a rate approximately 400‐fold higher than the combined incidence observed in the present adolescent cohort (0.100 per 1000 h) [12]. While methodological differences in injury definition and ascertainment may partially account for this discrepancy, the magnitude of difference potentially suggests a protective effect of younger age on golf‐related injury risk. The lower injury burden in adolescent elite golfers may reflect reduced cumulative microtrauma, superior tissue healing capacity, lower body mass and associated biomechanical loads, and potentially more conservative training volumes compared to adult populations. However, longitudinal tracking of these athletes into adulthood would be necessary to determine whether early specialisation in golf confers lasting protection or merely delays injury onset until higher competitive levels are reached.

These observations align with previous research by Bueno et al. (2018), who reported a 2% injury rate among adolescent golfers [13]. However, other studies suggest higher rates; Fradkin et al. (2006) found that individuals aged 10–19 years accounted for 20% of golf‐related injuries presenting at emergency departments [14]. This discrepancy may stem from variations in study design, participant populations, and injury types considered [14]. The current study focuses on self‐reported injuries, while Fradkin et al. (2006) evaluated emergency department records, capturing more severe injuries. Furthermore, studies on specific injury locations, such as back pain, report higher incidence rates; Quinn et al. (2022) found a 41% incidence of low back pain among young male elite golfers [15]. This highlights the importance of considering injury location when comparing injury rates across studies.

The findings from this study reveal a relatively low injury incidence rate compared to other sports, with only 8 of the 56 participants reporting golf‐related injuries. The annual incidence rates of 0.229, 0.624, and 0.100 per 1000 h suggest that golf may pose a lower risk of injury than other sports, such as soccer, which has injury rates as high as 7.21 per 1000 h [16]. While the overall injury rate appears low, the data indicates a concerning trend: 30.36% of participants reported at least one injury in the previous year, with many occurring during other sports rather than golf. However, this rate is still lower than the injury prevalence of 34.1% to 65% reported among adolescent athletes [17, 18]. This suggests that while adolescents engaged in golf do experience injuries, the overall incidence is notably less than in other sports.

This investigation uncovers a distinctive injury profile among elite adolescent golfers, contrasting with adult golfers and adolescents in other sports. While adult amateur golfers commonly sustain lower back injuries (18.3%–36.4%) [4, 12], this study identified mid back (thoracic spine) injuries as the most frequent among adolescent golfers (47.06%), followed by ankle, lower back, and knee injuries (29.41% and 23.52%, respectively). Mid back injuries are infrequently emphasised in adult golfer data [4], highlighting the unique injury patterns in this adolescent population. Additionally, while adolescent athletes in sports like basketball and soccer typically present with ankle sprains and knee injuries [16], this study indicates a significantly elevated incidence of mid‐back issues among young golfers. Consequently, the biomechanics of the golf swing during formative years may impose distinctive stress on the thoracic spine, emphasising the need for age‐specific injury prevention and management strategies for young athletes.

### 4.2 Training Volume and Injury Reporting

This study revealed that the volume of golf training did not correlate with injury incidence, contradicting the common finding that increased training load can lead to a higher risk of overuse injuries [6, 17]. This highlights the need for further investigation into the specific mechanisms of injury in golf and other factors that could influence injury risk. Comparisons with existing literature are challenging due to variability in methodologies for injury reporting and limited specific injury data for this demographic. Injury risk characterisation is articulated through various frameworks, employing either incidence or prevalence‐based measures. To facilitate comparisons and risk assessment, injury rates in this study were computed per 1000 player hours, as recommended in the International Olympic Committee Consensus Statement [11]. Many studies also encompass broader age ranges [15, 19, 20], complicating the ability to isolate injury rates specific to adolescents.

### 4.3 Injury Risk Factors

The lack of significant correlation between several key factors and injury risk in this elite adolescent golfing population presents a unique perspective given the limited research focusing on this cohort. Notably, the absence of statistically significant correlations between injury risk and factors such as age, gender, warm‐up practices, engagement in other sports, professional instruction, and golf club fitting suggests that these commonly assumed risk factors may not play a critical role in determining injury outcomes for adolescent golfers.

The literature often suggests that gender can influence injury risk [19, 21]. For example, a study on adult amateur golfers found higher injury rates in men, particularly regarding back pain [19]. Conversely, studies on adolescent athletes typically indicate females are at a higher risk of injury [18, 21]. This survey study did not observe a significant gender difference in injury risk among elite adolescent golfers, possibly due to high training levels and better access to resources that may mitigate gender‐related injury risks.

The lack of significant correlations also contradicts studies highlighting the importance of warm‐up routines and engagement in multiple sports as protective factors against injuries. Previous research suggests proper warm‐up practices can mitigate injury risks [16], while participation in various sports may distribute physical stress across different muscle groups and joints, reducing overuse injuries [22]. A substantial portion of injuries reported (46.06%) occurred during non‐golf activities, supporting the notion that adolescent athletes face cumulative injury risks across various sports [17]; however, it opposes literature asserting that early specialisation heightens injury risk [23]. This suggests that other factors, not examined in this study, may play a more prominent role in injury occurrence. This may include biomechanical factors involving individual variations in swing mechanics [24], flexibility, strength, and physical maturity [25]; psychological factors such as stress levels, competitive pressure, early sport specialisation, and training burnout [26]; environmental factors such as playing conditions [27], and equipment maintenance.

This research reinforces a documented correlation between prior injuries and an elevated likelihood of subsequent injuries in adolescent athletes [28], with 17.65% of documented injuries classified as relapses. Past studies indicate that athletes with prior injuries are at a markedly higher risk for subsequent injuries, with odds ratios suggesting a nearly fourfold increase in risk [29]. This suggests that injury recurrence is a critical consideration in adolescent sports, particularly in physically demanding activities like golf. Furthermore, traumas sustained during adolescence may precipitate future injuries, as studies suggest that knee injuries in adolescents heighten the probability of developing knee osteoarthritis in adulthood [30]. Longitudinal investigations with follow‐up into adulthood are imperative to elucidate the lasting health consequences of athletic injuries sustained during adolescence.

The findings raise questions about the effectiveness of rehabilitation and monitoring strategies for adolescent golfers. Given that some reported injuries were recurrences, it highlights the necessity for tailored rehabilitation programs addressing the needs of young athletes with a history of injury. The lack of significant correlations between various risk factors further complicates the understanding of injury recurrence. While some studies suggest that proper warm‐up routines and varied sports participation can serve as protective factors, the current findings imply these measures may not be as effective in preventing recurrences among elite adolescent golfers. Overall, while the study's findings contribute valuable insights into the injury profiles of adolescent golfers, they also highlight the complexities of injury risk factors and the necessity for tailored injury prevention strategies that consider the unique characteristics of the sport [31].

## 5 Limitations

Some limitations need to be acknowledged. Firstly, the cross‐sectional design restricts the ability to draw definitive conclusions regarding the temporal relationships between risk factors and injury occurrences. This snapshot nature limits the assessment of how changes in training volume or other variables may influence injury risk. Longitudinal studies are warranted to reveal the dynamic interplay between these factors and provide a comprehensive understanding of injury development in elite adolescent golfers.

Secondly, reliance on self‐reported data constitutes a methodological weakness that introduces multiple sources of bias and measurement error. Self‐report bias represents a threat to data validity, as participants may inaccurately recall injury details or misclassify injury severity. The retrospective 12‐month recall period exacerbates this limitation, as memory decay increases with temporal distance from the injury event, potentially leading to systematic underreporting of minor injuries or overemphasis on more severe incidents. Adolescent participants, given their developmental stage and potentially limited medical knowledge, may struggle to accurately identify, characterise, or differentiate between injury types, further compromising data accuracy. This is particularly problematic when distinguishing between acute traumatic injuries and chronic overuse conditions, or when determining whether an injury was directly golf‐related or attributable to other activities. The absence of objective verification through medical records, clinical examination, or injury surveillance systems represents a weakness that undermines confidence in the reported injury incidence rates and characteristics. Future investigations should incorporate prospective injury surveillance with objective clinical assessment and medical documentation to enhance data reliability and validity, thereby strengthening the evidentiary foundation for clinical recommendations.

Further, the small sample size (*n* = 56) may present constraints on generalisability, for example the statistical power to detect significant associations between injury risk factors and occurrences is limited. Post‐hoc power analysis suggests that the study was underpowered to identify small to moderate effect sizes, which may explain the absence of statistically significant relationships between variables such as training volume, gender, and injury risk. Consequently, the null findings should be interpreted with caution, as they may reflect Type II error rather than genuine absence of association. In addition, the exclusive recruitment of participants from a single talent development programme in Western Australia severely restricts the external validity of the results, precluding confident generalisation to the broader population of elite adolescent golfers nationally or internationally. The homogeneity of the sample, while beneficial for internal consistency, may mask important variations in injury patterns that exist across different geographical regions, training environments, and competitive levels. These sampling limitations fundamentally weaken the evidentiary strength of the study's conclusions and necessitate replication with substantially larger, more diverse cohorts before definitive statements regarding injury risk factors in this population can be made.

Lastly, the deliberate decision to omit standardised definitions for ‘injury,’ ‘golf‐related injury,’ and ‘non‐golf‐related injury’ represents a methodological weakness that compromises the validity and comparability of the findings. While this decision was made pragmatically to reduce participant burden and accommodate the cognitive developmental range of 10 to 19‐year‐old respondents, it introduces measurement error and threatens both internal and external validity. Without operationalised definitions, participants likely applied heterogeneous, idiosyncratic interpretations when classifying their injuries, potentially resulting in systematic misclassification bias. For instance, some participants may have reported only injuries requiring medical attention, while others may have included minor discomfort or transient pain. Similarly, the classification of injuries as ‘golf‐related’ versus ‘non‐golf‐related’ may have varied considerably based on individual interpretation, particularly for overuse injuries with multifactorial aetiologies. This lack of standardisation fundamentally weakens the study by precluding meaningful comparison with existing literature that employs consensus injury definitions (e.g., time‐loss definitions, medical attention definitions). It also limits the reproducibility of the research and the ability to aggregate findings across studies for meta‐analysis. This methodological decision, while expedient, represents a trade‐off that prioritised survey completion rates over data quality and scientific rigour. Future research should employ validated, age‐appropriate injury definitions aligned with international consensus statements to ensure methodological robustness and facilitate evidence synthesis across studies.

In summary, while this study provides valuable insights into the injury profiles of elite adolescent golfers, the limitations discussed highlight the need for caution in interpreting results. The study's primary contribution lies in identifying the need for more rigorous epidemiological research in this population, rather than providing robust evidence for specific injury prevention interventions. Addressing these limitations in future research will be crucial for advancing the understanding of injury patterns and risk factors within this population, ultimately informing targeted interventions and strategies for injury prevention and management.

## 6 Practical Implications

- Injury Rates: Elite adolescent golfers experience relatively low injury rates compared to athletes in more physically demanding sports, with many injuries occurring during non‐golf activities rather than while playing golf itself.
- Common Injuries: The most frequently reported injuries among young golfers include mid back, ankle, and lower back injuries, highlighting the need to pay attention to specific body areas during training and competition.
- Risk Factors Uncertain: Factors commonly believed to influence injury risk, such as age, gender, and training volume, did not show a significant connection to injuries in this group. However, the small sample size (*n* = 56) limited detection of small‐to‐moderate effects, suggesting null findings may represent Type II error rather than true absence of relationships.
- Injury Recurrence: A notable number of injuries reported were relapses of previous injuries, indicating that young athletes with a history of injury may need special attention in their recovery and rehabilitation processes.
- Importance of Warm‐Up: Many young golfers engage in warm‐up exercises before playing, which is a positive practice that can help prepare their bodies for activity, though it did not show a direct link to reducing injury risk in this study.

## 7 Conclusion

This study examined injury patterns among elite adolescent golfers (*n* = 56), revealing a 30.36% injury prevalence with incidence rates of 0.229, 0.624, and 0.100 per 1000 h for practice, competition, and combined activities, respectively. Mid‐back injuries were most prevalent (47.06%), followed by ankle (29.41%), lower back (29.41%), and knee (23.52%) injuries, contrasting with adult golfer profiles where lower back injuries predominate. Notably, 52.94% of injuries occurred during non‐golf activities, and 17.65% were recurrences. No significant associations were identified between injury risk and age, gender, training volume, warm‐up practices, professional instruction, or club fitting, though the limited sample size may have constrained statistical power to detect meaningful relationships. These findings highlight the unique injury profile of adolescent golfers and underscore the need for age‐specific injury prevention strategies. Future research with larger, more diverse samples and prospective designs is warranted to validate these findings and elucidate injury risk factors in this population.

## Author Contributions

**Stephen Lee:** conceptualization, methodology, software, investigation, validation, resources, writing – review and editing, writing – original draft, project administration, visualization. **Michele Lastella:** writing – review and editing, supervision. **Andrew Vitiello:** writing – review and editing, supervision. **Henry Pollard:** writing – review and editing, conceptualization, methodology, supervision.

## Transparency Statement

The lead author Stephen Lee affirms that this manuscript is an honest, accurate, and transparent account of the study being reported; that no important aspects of the study have been omitted; and that any discrepancies from the study as planned (and, if relevant, registered) have been explained.

## Data Availability

The authors have nothing to report.
